# A theoretical framework to analyse the flow of particles in a dynamical system with stochastic transition rates and site capacities

**DOI:** 10.1098/rsos.220698

**Published:** 2022-10-19

**Authors:** Aditi Jain, Arun Kumar, Arvind Kumar Gupta

**Affiliations:** Department of Mathematics, Indian Institute of Technology Ropar, Rupnagar, 140001 Punjab, India

**Keywords:** ribosome flow model with different site sizes, stochasticity, random variables, random matrix theory

## Abstract

We study the stochasticity in a dynamical model: ribosome flow model with different site sizes that models the unidirectional movement of particles controlled by transition rates along a lattice having different site sizes. Our work models the parameters as random variables with known distributions and investigates the steady-state flow rate under this notion by using tools from the random matrix theory. Some closed-form theoretical results are derived for the steady-state flow rate under some restrictive assumptions such as random variables being independent and identically distributed. Furthermore, for arbitrary but bounded stochastic transition rates, stochastic site capacities, or both, we establish bounds for the steady-state flow rate. Our analysis can be generalized and applied to study the flow of particles in numerous transport systems in the stochastic environment.

## 1. Introduction

There are many natural or man-made transport processes that can be viewed as non-equilibrium systems where ‘particles’ move along a one-dimensional lattice of ordered ‘sites’. These processes include movement of molecular motors [1], gene translation [2], transcription [3], intracellular transport [4], vehicular traffic [5], phosphorelay [6] and many more. A basic physical concept in these transport processes is the simple exclusion principle which asserts that each site along the lattice can be occupied by at most one particle. Several particles move simultaneously along the same track thus forming traffic congestion behind a particle that does not move for a long time [7].

In reality, there are various types of stochasticity present in all these systems due to several reasons such as intrinsic factors, experimental noise, uncertainty, etc. For example, in many cellular transport processes, chemical reactions are stochastic due to their microscopic nature where the chemical transitions take place with exponentially distributed waiting times [8]. Therefore, it is necessary to speculate these transport processes in the stochastic environment to understand them in a better way. A key factor to analyse is the flow rate and hence it is of considerable interest to understand how this rate is affected under stochasticity in these processes. Generally, the stochastic effects are modelled by considering the parameters drawn from the probability distributions following certain physical arguments.

Mathematical models are becoming increasingly significant in understanding the particle flow dynamics because they can be used to make qualitative and quantitative predictions about the effects of changing parameters on system dynamics [9–15]. An elementary model from statistical physics based on the simple exclusion principle is the stochastic model called the totally asymmetric simple exclusion process (TASEP) [16–18]. Particles hop unidirectionally with some probability from one site to the next free site in this fundamental model, illustrating that the particles have volume and cannot pass one another. As a result, each site can be either occupied by a single particle or remain vacant, meaning that all sites are of equal size.

TASEP and its variants have been extensively used to model a variety of processes such as cellular cargo transport, pedestrian dynamics, transport across biomembrane channels, the packets flow in communication networks, and many more [19]. However, due to the indirect interactions between the particles, analysis of TASEP is cumbersome and in particular, results for TASEP hold when the number of sites goes to infinity. In particular simplified cases, such as the model with homogeneous rates, exact solutions can be obtained, whereas in the non-homogeneous stochastic case, one looks for extensive and time-consuming Monte Carlo simulations.

A deterministic mathematical model called the ribosome flow model (RFM) which is derived as a dynamical mean-field approximation of TASEP was introduced by Reuveni *et al.* [20]. This model is easy to simulate and highly amenable to mathematical analysis using tools from the theory of cooperative dynamical systems [21], contraction theory [22] and Perron–Frobenius theory [23]. The RFM includes *n* consecutive sites along a one-dimensional track. The normalized occupancy level (or density) of site *i* at time *t* is described by a state variable $x_{i}(t)\;:\;R_{+}⟶[0,1]$ where *x*_*i*_(*t*) = 0[*x*_*i*_(*t*) = 1] means that site *i* is completely free [full] at time *t*. The flow between site *i* and site *i* + 1 is controlled by the transition parameter *λ*_*i*_ > 0. The dynamics of the RFM is given by the *n* nonlinear ordinary differential equations:

1.1 $${\dot{x}}_{i}=\lambda_{i-1}x_{i-1}(1-x_{i})-\lambda_{i}x_{i}(1-x_{i+1}),\;i=1,\ldots ,n,$$

where *x*_0_(*t*) := 1 and *x*_*n*+1_(*t*) := 0. Equation (1.1) depicts that the flow of particles from site *i* to site *i* + 1 is *λ*_*i*_*x*_*i*_(1 − *x*_*i*+1_). This model includes a ‘soft’ version of the simple exclusion principle i.e. the flow increases with the occupancy level at site *i* and it decreases as site *i* + 1 becomes full. The flow or output rate from the last site at time *t* is denoted by *R*(*t*) := *λ*_*n*_ *x*_*n*_(*t*). It has been proved that the steady state is always achieved by the dynamics [24]. Clearly, RFM is a nonlinear compartmental system where each state variable describes the amount of material in each compartment. Several generalizations of the RFM have been widely used to mimic the flow of ribosomes along an mRNA molecule or networks of mRNAs as well as other key cellular transport processes, etc. [25–32].

**Figure 1.**
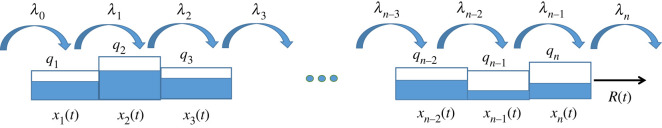
The representation of unidirectional flow along a chain of *n* sites having different sizes. The parameter *λ*_*i*_ > 0 controls the transition rate from site *i* to site *i* + 1. The parameter *q*_*i*_ represents the size of the site *i*. The state variable x_*i*_(*t*) ∈ [0, *q*_*i*_] represents the density at site *i* at time *t*. *R*(*t*) denotes the output rate from the lattice at time *t*.

The capacity of each site or compartment is taken to be equal in the RFM. However, each site can have a different size, i.e. capacity of two different compartments can be unequal. For example, consider the flow of vehicles down a road: depending on the number of parallel lanes, each section would have a different capacity. To incorporate this feature, a generalization of the RFM called ribosome flow model with different site sizes (RFMD) was introduced [33]. Each particle in this model hops forward to the next site not only if it is vacant, but also if it is ready to accept the particle. The dynamics of the RFMD is given by the set of *n* nonlinear ordinary differential equations:

1.2 $${\dot{x}}_{i}=\lambda_{i-1}x_{i-1}(q_{i}-x_{i})-\lambda_{i}x_{i}(q_{i+1}-x_{i+1}),\;i=1,\ldots ,n,$$

where *x*_0_(*t*) := 1 and *x*_*n*+1_(*t*) := 0. The state variable $x_{i}(t)\;:\;R_{+}⟶[0,q_{i}]$ describes the normalized occupancy level of site *i* at time *t* where *x*_*i*_(*t*) = 0[*x*_*i*_(*t*) = *q*_*i*_] means that site *i* is completely empty [full] (figure 1). The parameter *q*_*i*_ ∈ (0, 1] describes the maximal capacity at each site. We review the mathematical properties of the RFMD in the next section. In the particular case, when *q*_*i*_ = 1 for all *i*, RFMD becomes the RFM.

The transition rates that are considered fixed in the RFM generally vary depending upon the available resources, the presence of various noise sources, etc. For example, variance of elongation factors like EF-Tu, tRNA molecules, etc. leads to randomness in the transition parameters in the translation process. In a recent paper, variations in the rates of the RFM have been studied by assuming them as random variables [34]. Using tools from random matrix theory, it is proved that the steady-state output rate is bounded above and below by two random values and that both bounds converge to a constant value when the random variables are independent and identically distributed (i.i.d.) [34].

The RFMD is a more useful model for analysing the movement of particles along a one-dimensional track as it incorporates an important dynamical feature of different site sizes that do not exist in the RFM. Therefore, it is crucial to understand this model with variable rates due to various levels of stochasticity. In this paper, we characterize the notion of randomness in the RFMD in the sense that the parameters are random variables with known distributions and analyse the steady-state flow rate in the RFMD through a theoretical approach under this assumption. In particular, this also models the transport phenomena having stochastic variations in the transition rates and fixed site sizes. Our main results also include some closed-form results under restrictions such as rates are i.i.d. random variables.

The paper is organized as follows. The next section recalls the dynamical properties of the RFMD that are relevant in our context. Section 3 describes our main theoretical results. The next section includes all the proofs of the given results. The final section summarizes the findings and suggests several directions for further research.

## 2. Dynamical properties of the RFMD

Let $R_{++}^{n}$ denote the set of *n*-dimensional vectors with all entries positive. Consider an RFMD with dimension *n* having transition rates $\lambda \in R_{++}^{n+1}$ and site sizes *q* ∈ (0, 1]^*n*^. Let *x*(*t*, *a*) denote the solution of the RFMD at time *t* for the initial condition *x*(0) = *a*. The state variable *x*_*i*_(*t*) corresponds to the normalized occupancy level at site *i* at time *t*, so we always assume that *a* belongs to the state space:

$$C\;:=\{x\in R^{n}\;:\;\;x_{i}\in [0,q_{i}],\;i=1,2,\ldots ,n\}.$$

It has been proved that there exists a unique *e* ∈ *int*(*C*) such that for any initial condition in *C*, the solution belongs to *int*(*C*) for all *t* > 0 and lim_*t*→∞_ *x*(*t*, *a*) = *e* [33]. In other words, the rates and site sizes determine a unique steady-state *e*, and any solution arising from different initial conditions in *C* converges to it.

At the steady state, for *x* = *e*, the left-hand side of all the equations in (1.2) is zero, so

$$\begin{array}{rl} \lambda_{0}(q_{1}-e_{1}) & =\lambda_{1}e_{1}(q_{2}-e_{2})\\ & =\lambda_{2}e_{2}(q_{3}-e_{3})\\ & \;\;\vdots \\ & =\lambda_{n-1}e_{n-1}(q_{n}-e_{n})\\ & =\lambda_{n}e_{n}. \end{array}$$

To put it another way, at the steady state, the flow into and out of each site is equal. Let *R* := *λ*_*n*_*e*_*n*_ denote the steady-state flow or output rate. It is clear that obtaining the solution of non-linear equations in (1.2) is not straightforward in general. However, it has been recently proved in [33] that the steady-state flow rate can be obtained from the spectral properties of a suitable tridiagonal matrix. Define $A_{n}\;:\;R_{+}⟶R^{\left(n+2)\times (n+2\right)}$ by

$$A_{n}(r)\;:=\left[\begin{array}{ccccc} 0 & \lambda_{0}^{-1/2} & 0 & \ldots & 0\\ \lambda_{0}^{-1/2} & (1-q_{1})r & \lambda_{1}^{-1/2} & \ldots & 0\\ 0 & \lambda_{1}^{-1/2} & (1-q_{2})r & \ldots & 0\\ & & \vdots \\ 0 & \ldots & 0 & (1-q_{n})r & \lambda_{n}^{-1/2}\\ 0 & \ldots & 0 & \lambda_{n}^{-1/2} & 0 \end{array}\right].$$

The above matrix *A*_*n*_(*r*) has real eigenvalues due to the fact that it is symmetric. Further, each element of *A*_*n*_(*r*) is non-negative and *A*_*n*_(*r*) is irreducible, implying that it has a simple maximal positive eigenvalue for each *r* [23]. It was shown in [33] that there exists a unique value r* ∈ (0, ∞) such that

2.1 $$\sigma (A_{n}(r^{*}))=r^{*},$$

and the steady-state flow rate satisfies

2.2 $$R=\frac{1}{(\sigma \;\;{\left(A_{n}(r^{*}))\right)}^{2}}.$$

The spectral representation above shows that the steady-state flow rate in the RFMD depends on the transition rates and site capacities. This spectral representation has various useful theoretical implications. It has been used to obtain results on the sensitivity analysis and quasi-concavity of the steady-state flow rate. It also allows the upper and lower bounds of *R* to be determined when the rates are random variables with some known distributions using tools from probability theory as shown in the following sections.

The main results on the steady-state flow rate in the RFMD given random transition rates or random site capacities are presented in the next section.

## 3. Main results

Let (Ω,F,P) be a probability space and all random variables in the next subsections are defined on this common probability space. We describe random variable *X* as almost sure bounded if there exists 0 ≤ *c* < ∞ such that P[|X|≤c]=1. Let $M_{X}\;:=\inf_{c\geq 0}\;\{P[|X|\leq c]=1\}$ and $m_{X}\;:=\underset{c\geq 0}{\sup}\;\{P[c\leq |X|]=1\}$. Let $R_{\geq \delta }\;:=\{x\in R\;:\;\;x\geq \delta >0\}$. The steady-state flow rate in the *n*-site RFMD is denoted by *R*_*n*_.

We analyse the value of *R*_*n*_ given the random transition rates or the site capacities. In the first §3.1, we provide results on the value of *R*_*n*_ by assuming that the transition rates are random variables and the site capacities are deterministic. The second §3.2 deals with the case when the site capacities are random variables and the transition rates are deterministic. The last §3.3 analyses *R*_*n*_ given the variability in all the rates.

### 3.1. The RFMD with stochastic transition rates

In this subsection, we consider randomness only in the transition rates, i.e. we assume that the size of all the compartments is fixed and tackle stochasticity or uncertainties in the transition rates by assuming them as random variables with some known distributions. This situation may model, for example, variations in the speed of the vehicles due to different human behaviours along a multi-lane road where there is a change in the number of lanes along the road.

The random variable *Z* := *X*^−1/2^ is almost sure bounded for *X* supported on $R_{\geq \delta }$. We further examine the steady-state flow rate by investigating two cases: homogeneous and non-homogeneous site capacities. In the first case, we assume that all the site capacities are equal. This assumption is certainly restrictive and is required in order to derive some closed-form theoretical outcomes.

**Case 1.** The homogeneous compartment sizes (*q*_*i*_ = *q*)

Firstly, we consider an RFMD with all the *q*_*i*_’s equal and denote their common value as *q*. The following result assumes that the rates are i.i.d. random variables. ▪

**Theorem 3.1.**

*Assume that rates λ*_*i*_ *in the RFMD with dimension* *n* *are independent copies of a random variable* *X* *having support on* $R_{\geq \delta }$. *Then* $R_{n}\overset{p}{\to }q^{2}{\left(2M_{X^{-1/2}}\right)}^{-2}$ *as* *n* → ∞, *i.e*. *R*_*n*_ *approaches the value* $q^{2}{\left(2M_{X^{-1/2}}\right)}^{-2}$ *with probability one, as* *n* → ∞.

The above result states that as the dimension of the RFMD increases, the steady-state flow rate converges with unit probability to a fixed value depending upon the constant site size *q* and on the minimal possible value (with probability one) that the random variable *X* attains. Clearly, for *q* = 1, we retrieve the case of variability in the rates in the RFM [34].

**Example 3.2.**

**Figure 2.**
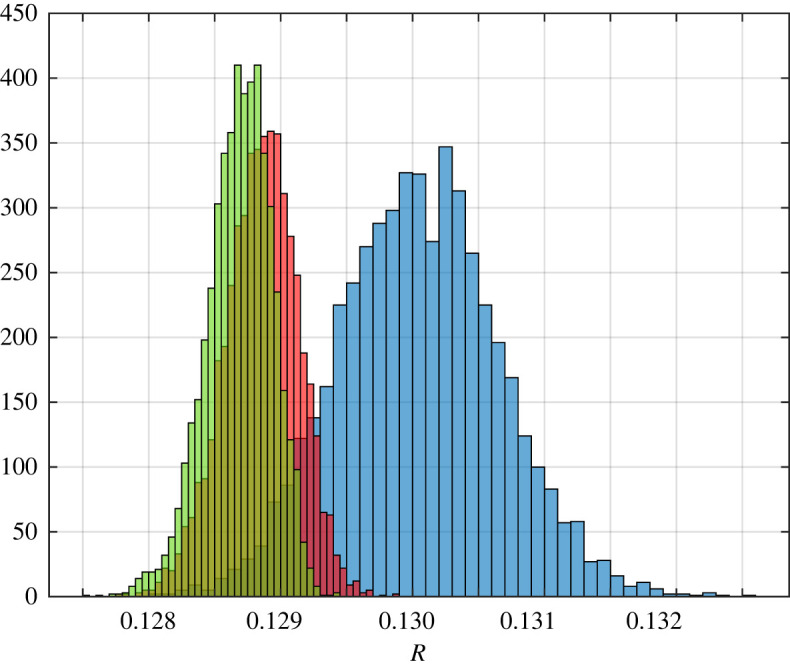
Histograms showing 5000 distinct values each for RFMD with dimensions 50, 500 and 1000 coloured in blue, red and green, respectively, for the steady-state flow rate in the RFMD with the parameters considered in example 3.2. Our theoretical result predicts that as *n* goes to infinity, the steady-state flow rate converges to 0.125 with probability one.

Suppose that *X* follows a half-normal distribution with mean having value 2 and standard deviation having value 0.1. Note that $M_{X^{-1/2}}=1/\sqrt{2}$. Let *q*_*i*_ = 0.5 for all *i*. In this case as *n* goes to infinity, $R_{n}\overset{p}{\to }0.125$ by theorem 3.1. A histogram of the results for *n* ∈ {50, 500, 1000} is shown in figure 2.

We now investigate the cases where the restrictive assumption of rates being i.i.d. random variables is a little bit relaxed. Our first case considers that the random variables *X*_*i*_ could be non-identical, but all independent and having the same support. In this case also, the analysis of the proof of theorem 3.1 proves that the steady-state flow rate asymptotically approaches the same value as in theorem 3.1. The next example exhibits this.

**Example 3.3.**

**Figure 3.**
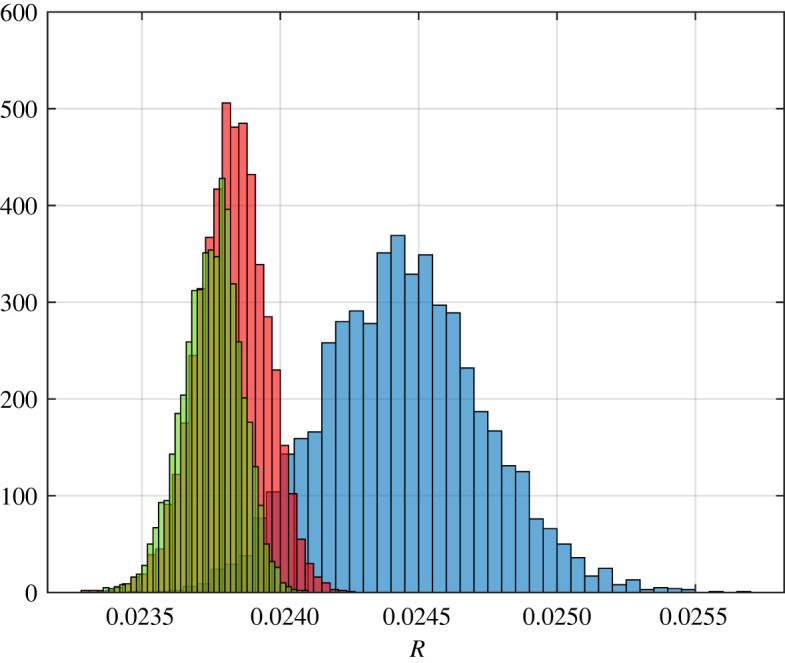
Histograms showing 5000 distinct values each for RFMD with dimension 50, 500 and 1000 coloured in blue, red and green, respectively, for the steady-state flow rate in the RFMD with the parameters considered in example 3.3. The theory predicts that as *n* goes to infinity, the steady-state flow rate converges to 0.0225 with probability one.

Let *n* + 1 independent random variables *X*_0_, *X*_1_, …, *X*_(*n*/2)−1_ be distributed using the half-normal distribution with mean having value 1 and standard deviation having value 0.1 and *X*_*n*/2_, *X*_(*n*/2)+1_,…, *X*_*n*_ distributed uniformly on [1, 2]. Note that $M_{X_{i}^{-1/2}}=1$ for all *i*. Let *q*_*i*_ = 0.3 for all *i*. The theory predicts that as *n* goes to infinity, $R_{n}\overset{p}{\to }0.0225$. A histogram of the results for *n* ∈ {50, 500, 1000} is depicted in figure 3.

In the second case, we allow a growing (but still tiny) number of random variables having support different from the other random variables. We use the notation *S*^*n*^ to denote the set of permutations on {1,2,…,n}. Consider a permutation *π* ∈ *S*^*n*^, and let $Y^{\pi }≜\pi \circ Y=(Y_{\pi (1)},Y_{\pi (2)},\ldots ,Y_{\pi (n)})$. Let $Y_{i}^{\pi }$ denote the *i*th element in *Y*^*π*^. The next result is a generalization of the theorem stated for *q* = 1 in [34].

**Theorem 3.4.**

*Let* *d* = *d*(*n*) > 0 *be an integer which has the property* lim_*n*→∞_ *d*(*n*)/*n* = 0. ${\left\{X_{i}\right\}}_{i=0}^{n-d}$ *is a collection of* (*n* − *d* + 1) *independent random variables having support on* $R_{\geq \delta }$, *and satisfies*

3.1 $$M_{X_{0}^{-1/2}}=M_{X_{1}^{-1/2}}=\cdots =M_{X_{n-d}^{-1/2}}.$$

${\left\{X_{i}\right\}}_{n-d+1}^{n}$ *is a collection of* *d* *random variables having support on the positive semi-axis and satisfies*

3.2 $$M_{X_{j}^{-1/2}}\leq \delta^{-1/2},\;j=n-d+1,\ldots ,n.$$

*Denote* $Y=(X_{0},X_{1},\ldots ,X_{n})$. *Fix a permutation* *π* ∈ *S*^*n*+1^. *Let each rate* *λ*_*i*_ *in the RFMD with dimension* *n* *be a copy of the random variable* $Y_{i}^{\pi }$, *for* 0 ≤ *i* ≤ *n* *and* *λ*_*i*_’*s are independent. Then* $R_{n}\overset{p}{\to }q^{2}{\left(2M_{X_{0}^{-1/2}}\right)}^{-2}$ *as* *n* → ∞.

The above result shows that the steady-state flow rate asymptotically approaches the same value as in theorem 3.1. We shall now describe results on the steady-state flow rate given the non-homogeneous site capacities.

**Case 2.**

The non-homogeneous compartment sizes

Secondly, we consider the case when some (or all) *q*_*i*_’s are non-identical. Let *q*_ℓ_ and *q*_*L*_ denote the minimum and maximum value of *q*, respectively. For given \epsilon > 0, define $a(\epsilon )\;:=P\{X^{-1/2}\geq M_{X^{-1/2}}-\epsilon \}$. The next result analyses the bounds of *R*_*n*_ for the finite dimension *n* of the RFMD. ▪

**Theorem 3.5.**

*Assume that rates* *λ*_*i*_ *in the RFMD are independent copies of a random variable* *X* *having support on* $R_{\geq \delta }$. *Consider two sequences of positive integers* (*n*_*i*_) *with* *n*_*j*_ < *n*_*i*_ for *j* < *i* *and* (*s*_*i*_) *with* *s*_*j*_ < *s*_*i*_ for *j* < *i* *and satisfying* *s*_*i*_ < *n*_*i*_ − 1 *for all* *i*. *Also, consider a decreasing sequence of positive scalars* *ε*_*i*_, *with* *ε*_*i*_ → 0. *Then* $R_{n_{i}}$ *in the RFMD with dimension* *n*_*i*_ *for any* *i* *satisfies*

3.3 $${\left(q_{\ell }\right)}^{2}{\left(2M_{X^{-1/2}}\right)}^{-2}\leq R_{n_{i}}\leq {\left(q_{L}\right)}^{2}{\left(2M_{X^{-1/2}}\right)}^{-2}(1+O(\epsilon_{i}+s_{i}^{-2})),$$

*with probability at least*

3.4 $$1-\exp\left(-\left\lfloor \frac{n_{i}-2}{s_{i}}\right\rfloor {\left(a(\epsilon_{i})\right)}^{s_{i}}\right).$$

The following general result examines the case where we have bounded but arbitrary *X*_*i*_’s. The set of all possible *s* consecutive integers from the set {1,2,…,n−1} is denoted by $J_{s}^{n-1}$.

**Theorem 3.6.**

*Assume that each rate* *λ*_*i*_ *in the RFMD with dimension* *n* *is a copy of a random variable* *X*_*i*_ *having support on* $R_{\geq \delta_{i}}$, *for* 0 ≤ *i* ≤ *n*. *Then* *R*_*n*_ *satisfies*

3.5 $${\left(q_{\ell }\right)}^{2}(\max_{1\leq i\leq n}X_{i-1}^{-1/2}+X_{i}^{-1/2}{)}^{-2}\leq R_{n}\leq {\left(q_{L}\right)}^{2}{\left(2\max_{1\leq s\leq n-1}\cos\left(\frac{\pi }{s+2}\right)\max_{J_{s}\in J_{s}^{n-1}}{\text{min}}_{i\in J_{s}}X_{i}^{-1/2}\right)}^{-2},$$

*with probability one.*

The above result shows that the steady-state flow rate may not approach a deterministic value in this scenario, but rather it is constrained by two random values above and below. Given several possible distributions of random transition rates, this result gives a notion of a range of output rates. The steady-state flow rate is examined in the next subsection, given deterministic transition rates and variability in site capacities.

### 3.2. The RFMD with stochastic compartment sizes

In this subsection, we consider randomness only in the site sizes, i.e. we assume that the transition rates are fixed and tackle fluctuations in the size of compartments by assuming them as random variables with some known distributions. This may model processes like the packets flow in communication networks. In the context of linear communication networks, the data packets are the moving particles and buffers are the sites [35]. Owing to many reasons such as run-down communication infrastructure or interference, there could be fluctuations in the capacity of the buffers holding the data packets [36]. The approach used here can be generalized to analyse the flow of packets in such networks.

We further analyse the steady-state flow rate by considering the cases of homogeneous and non-homogeneous transition rates. We assume that all the transition rates are equal in the first case. Of course, this assumption is limiting, yet it is required to derive some closed-form theoretical results. However, it has some empirical support, for example, the rate of data packet transmission in a communication network can be the same under similar conditions.

**Case 1.**

The homogeneous transition rates (*λ*_*i*_ = *λ*)

Firstly, we consider the case when all the *λ*_*i*_’s are equal and denote their common value by *λ*. The next result assumes that the site capacities are i.i.d. random variables. ▪

**Theorem 3.7.**

*Assume that site capacities* *q*_*i*_ *in the RFMD with dimension* *n* *are independent copies of a random variable* *Q* *having support on* [*β*, *γ*] *where* 0 < *β* < *γ* ≤ 1. *Then* $R_{n}\overset{p}{\to }{\left(m_{Q}\right)}^{2}\lambda /4$ *as* *n* → ∞.

The above result states that the steady-state flow rate asymptotically approaches a constant value with probability one that depends on the constant transition rate *λ* and the minimal possible value (with probability one) of the random variable *Q* as the RFMD’s dimension grows.

**Example 3.8.**

**Figure 4.**
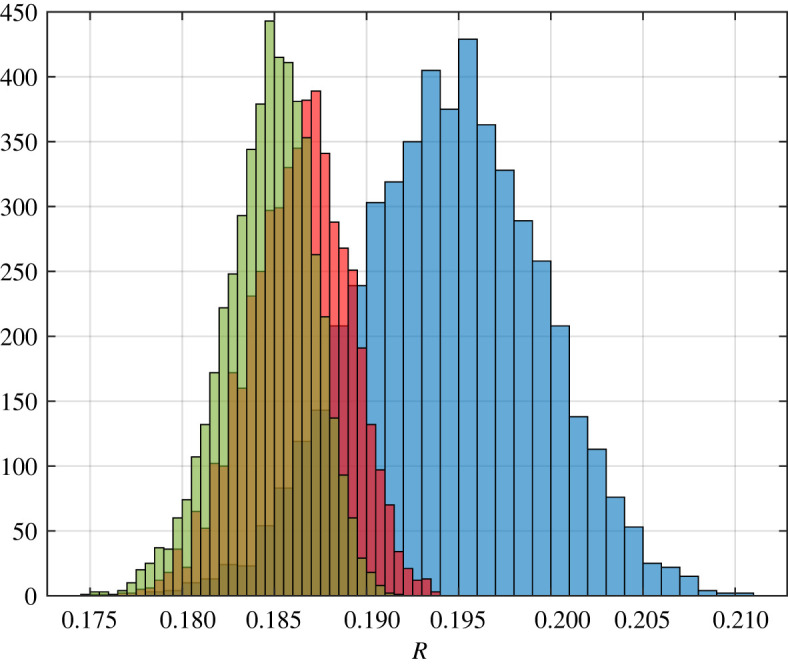
Histograms showing 5000 distinct values each for RFMD with dimensions 50, 500 and 1000 coloured in blue, red and green, respectively, for the steady-state flow rate in the RFMD with the parameters considered in example 3.8. Our theoretical result shows that as *n* goes to infinity, the steady-state flow rate converges to 0.16 with probability one.

Assume that *Q* has a uniform distribution on the range [0.8, 1]. Take note of *m*_*Q*_ = 0.8. For all *i*, let *λ*_*i*_ = 1. In this case, theorem 3.7 implies that as *n* approaches infinity, $R_{n}\overset{p}{\to }0.16$. A histogram of the results for *n* ∈ {50, 500, 1000} is shown in figure 4.

Likewise, in the first subsection, we now examine the cases where we allow some relaxations in the assumption that the site capacities are i.i.d. random variables. Our first case considers the random variables *Q*_*i*_ that might be non-identical, but all independent and they all have the same minimum bound. The following example shows that if each site capacity *q*_*i*_ is taken from the *Q*_*i*_ distribution, then again the steady-state flow rate approaches the same value as given in theorem 3.7.

**Example 3.9.**

**Figure 5.**
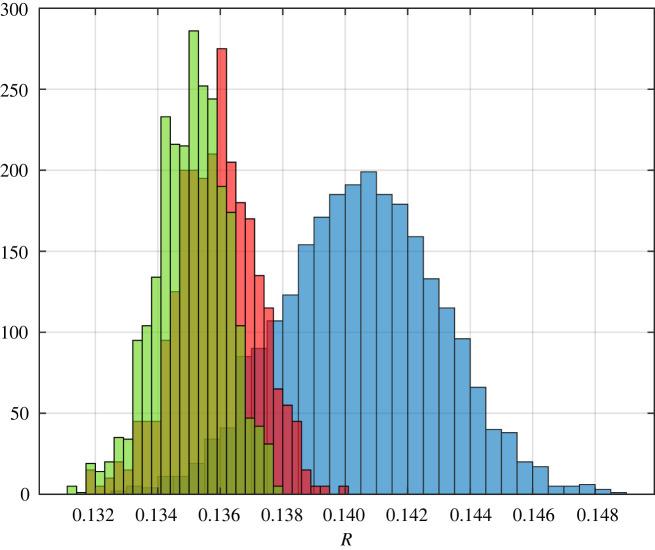
Histograms showing 2500 distinct values each for RFMD with dimension 50, 500 and 1000 coloured in blue, red and green, respectively, for the steady-state flow rate in the RFMD with the parameters considered in example 3.9. The theory forecasts that as *n* goes to infinity, the steady-state flow rate converges to 0.125 with probability one.

Let *n* independent random variables *Q*_1_, …, *Q*_*n*/2_ distributed uniformly on [0.7, 0.9] and *Q*_(*n*/2)+1_,…, *Q*_*n*_ distributed uniformly on [0.7, 0.8]. Note that $m_{Q_{i}}=0.7$ for all *i*. Let *λ*_*i*_ = 1 for all *i*. Thus, our theory predicts that as *n* approaches infinity, $R_{n}\overset{p}{\to }0.125$. A histogram of the results for *n* ∈ {50, 500, 1000} is depicted in figure 5.

We consider an increasing (but small in comparison to *n*) number of random variables modelling site sizes to have a different support than the rest of the other random variables in the next result.

**Theorem 3.10.**

*Consider an integer* *d* = *d*(*n*) > 0 *with the property* lim_*n*→∞_*d*(*n*)/*n* = 0. ${\left\{Q_{i}\right\}}_{i=1}^{n-d}$ *is a collection of* (*n* − *d*) *independent random variables having support on* [*β*, *γ*] *where* 0 < *β* < *γ* ≤ 1 *and satisfies*

3.6 $$m_{Q_{1}}=m_{Q_{2}}=\cdots =m_{Q_{n-d}}.$$

${\left\{Q_{i}\right\}}_{n-d+1}^{n}$ *is a collection of* *d* *random variables having support on* [*μ*_*i*_, *τ*_*i*_] *where* 0 < *μ*_*i*_ < *τ*_*i*_ ≤ 1 *for* i=n−d+1,…,n *and satisfies*

3.7 $$m_{Q_{j}}\geq \beta ,\;j=n-d+1,\ldots ,n.$$

*Denote* *Y* = (*Q*_1_, *Q*_2_, …, *Q*_*n*_). *Consider a permutation* *π* ∈ *S*^*n*^. *Each site capacity* *q*_*i*_ *in the RFMD with dimension* *n* *is a copy of the random variable* $Y_{i}^{\pi }$, *for* 1 ≤ *i* ≤ *n* *and* *q*_*i*_’*s are independent. Then* $R_{n}\overset{p}{\to }{\left(m_{Q_{1}}\right)}^{2}\lambda /4$ as *n* → ∞.

The above result shows that the steady-state flow rate approaches the same value as *n* goes to infinity as in theorem 3.7. Next, we shall discuss the case of non-homogeneous transition rates.

**Case 2.**

The non-homogeneous transition rates

Secondly, we consider the case when some (or all) *λ*_*i*_’s are non-identical. Let *λ*_ℓ_ and *λ*_*L*_ denote the minimum and maximum value of $\left\{\lambda_{i}^{-1/2}\;:\;\;i=0,1,\ldots ,n\right\}$, respectively. For *η* > 0, define $b(\eta )\;:=P\{Q\leq m_{Q}+\eta \}$. The following result provides the bounds of *R*_*n*_ for the finite dimension *n* of the RFMD. ▪

**Theorem 3.11.**

*Let us assume that site capacities* *q*_*i*_ *in the RFMD are independent copies of a random variable* *Q* *having support on* [*β*, *γ*] *where* 0 < *β* < *γ* ≤ 1. *Choose two positive integer sequences* (*n*_*i*_) *with* *n*_*j*_ < *n*_*i*_ *for* *j* < *i* *and* (*s*_*i*_) *with* *s*_*j*_ < *s*_*i*_ *for* *j* < *i* *and satisfying* *s*_*i*_ < *n*_*i*_ *for all* *i*, *and a decreasing sequence of positive scalars* *η*_*i*_, *with* *η*_*i*_ → 0. *Then* $R_{n_{i}}$ *in the RFMD with dimension* *n*_*i*_ *for any* *i* *satisfies*

3.8 $${\left(m_{Q}\right)}^{2}{\left(2\lambda_{L}\right)}^{-2}\leq R_{n_{i}}\leq {\left(m_{Q}\right)}^{2}{\left(2\lambda_{\ell }\right)}^{-2}\left(1+\frac{\eta_{i}^{2}}{m_{Q}}+\frac{2\eta_{i}}{m_{Q}}\right)(1+O(s_{i}^{-2})),$$

*with probability at least*

3.9 $$1-\exp\left(-\left\lfloor \frac{n_{i}-1}{s_{i}}\right\rfloor {\left(b(\eta_{i})\right)}^{s_{i}}\right).$$

Notably, the convergence rate (3.8) to the value given for homogeneous transition rate in theorem 3.7 as *n* increases is slower than the rate of convergence (3.3) to the value given for homogeneous site size in theorem 3.1. The following result deals with the situation where *Q*_*i*_’s are arbitrary yet bounded. They do not have to be independent or identical.

**Theorem 3.12.**

*Suppose that every site capacity* *q*_*i*_ *in the RFMD with dimension* *n* *is a copy of a random variable* *Q*_*i*_ *having support on* [*β*_*i*_, *γ*_*i*_] *where* 0 < *β*_*i*_ < *γ*_*i*_ ≤ 1, *for* 1 ≤ *i* ≤ *n*. *Then* *R*_*n*_ *satisfies*

3.10 $${\left({\text{min}}_{1\leq i\leq n}Q_{i}\right)}^{2}{\left(2\lambda_{L}\right)}^{-2}\leq R_{n}$$

*and*

3.11 $$\max_{1\leq s\leq n}(2\lambda_{\ell }\cos\left(\frac{\pi }{s+1}\right)+\max_{J_{s}\in J_{s}^{n}}(1-\max_{i\in J_{s}}Q_{i})\frac{1}{{\left(R_{n}\right)}^{1/2}})\leq \frac{1}{{\left(R_{n}\right)}^{1/2}}.$$

The above theorem shows that steady-state flow rate is explicitly bounded below by a random quantity and the other bound follows an implicit relationship. In the above subsections, we derive the theoretical results where we allow assumptions on the transition rates as random variables and the site capacities are deterministic and vice-versa. However, in the following subsection, we provide the bounds for the steady-state flow rate in the most general scenario, when the capacities of the sites and the values of the transition rates are random.

### 3.3. The stochastic RFMD

We state our last result where we assume that all the parameters are arbitrary random variables but bounded and they need not be independent or identical. For $J_{s}\in J_{s}^{n-1}$, let *H*_*s*_ be the set $\left\{J_{s}\cup (\ell (J_{s})+1)\right\}$ where ℓ(*J*_*s*_) denotes the last entry of the set *J*_*s*_.

**Theorem 3.13.**

*Assume that each rate* *λ*_*i*_ *in the RFMD with dimension* *n* *is a copy of a random variable* *X*_*i*_ *having support on* $R_{\geq \delta_{i}}$, *for* 0 ≤ *i* ≤ *n*. *Suppose that each site capacity* *q*_*i*_ *in the RFMD is a copy of a random variable* *Q*_*i*_ *having support on* [*β*_*i*_, *γ*_*i*_] *where* 0 < *β*_*i*_ < *γ*_*i*_ ≤ 1, *for* 1 ≤ *i* ≤ *n*. *Then* *R*_*n*_ *satisfies*

3.12 $${\left({\text{min}}_{1\leq i\leq n}Q_{i}\right)}^{2}(\max_{1\leq i\leq n}X_{i-1}^{-1/2}+X_{i}^{-1/2}{)}^{-2}\leq R_{n}$$

*and*

3.13 $$\max_{1\leq s\leq n-1}\max_{J_{s}\in J_{s}^{n-1}}\left(2\cos\left(\frac{\pi }{s+2}\right){\text{min}}_{i\in J_{s}}X_{i}^{-1/2}+(1-\max_{i\in H_{s}}Q_{i})\frac{1}{{\left(R_{n}\right)}^{1/2}}\right)\leq \frac{1}{{\left(R_{n}\right)}^{1/2}}.$$

The above result states that steady-state flow rate is bounded by two random quantities: the lower bound is explicit and the other bound follows an implicit relationship. The theoretical result stated here is the most general result that holds for variability or fluctuations both in the transition rates and the site capacities. We state an example to demonstrate the above theorem.

**Example 3.14.**

**Figure 6.**
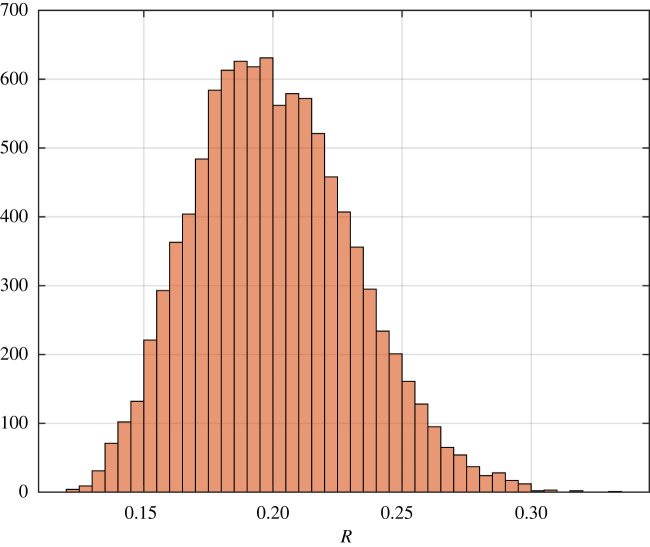
Histogram of 10 000 different values for RFMD with dimension 3 for the steady-state flow rate in the RFMD with the parameters considered in example 3.14. The theory predicts that the steady-state flow rate lies between 0.0625 and 0.49.

**Note**: Even though both the transition rates and the site capacities themselves are i.i.d. random variables, the steady-state flow rate does not converge to a deterministic value in this situation, contrary to our earlier theoretical conclusions. This can be explained as follows. Suppose that each rate *λ*_*i*_ is generated using the distribution of a random variable *X* that takes values in the interval [*a*, *b*], where *a* > 0. Consider that every site capacity *q*_*i*_ is selected using the distribution of a random variable *Q* taking values in the interval [*c*, *d*], where 0 < *c* < *d* ≤ 1. In this respect, we have

Consider an RFMD with dimension *n* = 3. Let the transition rates *λ* distributed uniformly on [1, 2] and the site sizes *q* distributed uniformly on [0.5, 0.7]. We have by calculation, 0.0625 ≤ *R*_*n*_ ≤ 0.49. Figure 6 depicts a histogram for *n* = 3.

3.14 $$\frac{1}{\sqrt{b}}\leq \frac{1}{\sqrt{X}}\leq \frac{1}{\sqrt{a}}$$

and

3.15 c≤Q≤d.

From equation (3.12), we have

3.16 $$\frac{c^{2}\;a}{4}\leq R_{n}.$$

From equation (3.13), we have

3.17 $$2\cos\frac{\pi }{s+2}\frac{1}{\sqrt{b}}+(1-d)\frac{1}{\sqrt{R_{n}}}\leq \frac{1}{\sqrt{R_{n}}}$$

and

3.18 $$\Rightarrow R_{n}\leq \frac{d^{2}\;b}{4\;(\cos{\left(\pi /(s+2))\right)}^{2}}.$$

As *n* → ∞, we can choose *s* large enough such that

3.19 $$R_{n}\leq \frac{d^{2}\;b}{4}.$$

Equations (3.16) and (3.19) imply

3.20 $$\frac{c^{2}\;a}{4}\leq R_{n}\leq \frac{d^{2}\;b}{4}.$$

Thus, the steady-state flow rate is bounded above and below by two different deterministic values as *n* → ∞.

## 4. Proofs

Firstly, we recall a result stated in [34] that will be used later on in proving theorem 3.1.

**Proposition 4.1.**

*Suppose that* ${\left\{U_{i}\right\}}_{i=1}^{n}$ *are i.i.d. random variables such that they are almost sure bounded. Let* *ε* > 0. *Consider an integer* 1 ≤ *s* ≤ *n* *and let* *S* *be the event that there exists an index* 1 ≤ *k* ≤ *n* − *s* + 1 *such that* $U_{k},\ldots ,U_{k+s-1}\geq M_{U_{1}}-\epsilon$. *Then the probability of* *S* *converges to one as* *n* → ∞.

**Proof.**

Let $f\;:=M_{U_{1}}-\epsilon$. For *k* ∈ {1, …, *n* − *s* + 1}, let *S*(ℓ) denote the event: *U*_ℓ_, …, *U*_ℓ+*s*−1_ ≥ *f*. Then

P(S)≥P(S(1)∪S(s+1)∪S(2s+1)∪⋯∪S(hs+1)),

where *h* is the largest integer such that (*h* + 1)*s* ≤ *n*. We have the i.i.d. *U*_*i*_’s and thus we get

$$P(S)\geq 1-(1-{\left(P(U_{1}\geq f)\right)}^{s}{)}^{h+1}.$$

Since the probability $P(U_{1}\geq f)$ is positive, when *n* → ∞, we have P(S)→1. ▪

**Proof of theorem 3.1.**

For ease of notation, let $Z_{i}\;:=\lambda_{i}^{-1/2}$ and each random variable *Z*_*i*_ is a copy of *X*^−1/2^. Then $A_{n}\;:\;R_{+}⟶R^{\left(n+2)\times (n+2\right)}$ can be written as

$$A_{n}(r)\;:=\left[\begin{array}{ccccc} 0 & Z_{0} & 0 & \ldots & 0\\ Z_{0} & (1-q)r & Z_{1} & \ldots & 0\\ 0 & Z_{1} & (1-q)r & \ldots & 0\\ & & \vdots \\ 0 & \ldots & 0 & (1-q)r & Z_{n}\\ 0 & \ldots & 0 & Z_{n} & 0 \end{array}\right].$$

The maximum eigenvalue of any symmetric matrix *A* having non-negative elements is bounded above by the maximum of the row sums of *A* [23], i.e.

4.1 $$\lambda_{\max}(A)\leq \max_{1\leq i\leq n}\sum_{\;j=1}^{n}a_{ij}.$$

Given *A*_*n*_(*r*) is a symmetric matrix with non-negative elements and hence,

4.2 $$\lambda_{max}(A_{n}(r))\leq \max_{1\leq i\leq n-1}(Z_{i}+Z_{i+1})+(1-q)r.$$

Also, we have $Z_{i}\leq M_{Z_{i}}$ for all *i*, which implies

4.3 $$\lambda_{\max}(A_{n}(r))\leq 2M_{Z_{1}}+(1-q)r,$$

with probability one. By equations (2.1) and (2.2), we get

4.4 $$\frac{q^{2}}{{\left(2M_{Z_{1}}\right)}^{2}}\leq R_{n}.$$

Let *G*_*s*_ denote the (*s* + 1) × (*s* + 1) symmetric tridiagonal matrix

$$G_{s}\;:=\left[\begin{array}{ccccc} 0 & 1 & 0 & \ldots & 0\\ 1 & 0 & 1 & \ldots & 0\\ 0 & 1 & 0 & \ldots & 0\\ & & \vdots \\ 0 & \ldots & 0 & 0 & 1\\ 0 & \ldots & 0 & 1 & 0 \end{array}\right].$$

It is known that the maximal eigenvalue of the above matrix is *λ*_max_(*G*_*s*_) = 2cos (*π*/(*s* + 2)) [37]. Let $f\;:=M_{Z_{1}}-\epsilon$. By proposition 4.1, we have an index *k* such that $Z_{k},\ldots ,Z_{k+s-1}\geq f$. We shall consider *k* = 1 and the other cases can be handled similarly. Let *B*_*n*_(*r*) be the matrix obtained by replacing the (*s* + 1) × (*s* + 1) principal minor corresponding to the indices 2, 3, …, *s* + 2 of *A*_*n*_(*r*) by *fG*_*s*_ + (1 − *q*)*r I*_*s*+1_. Hence *B*_*n*_(*r*) is given by

$$\left[\begin{array}{cccccccc} 0 & Z_{0} & 0 & 0 & \ldots & \ldots & \ldots & 0\\ Z_{0} & (1-q)r & f & 0 & \ldots & \ldots & \ldots & 0\\ 0 & f & (1-q)r & f & 0 & \ldots & \ldots & 0\\ & & \ddots \\ 0 & \ldots & 0 & f & (1-q)r & Z_{s+1} & \ldots & 0\\ 0 & \ldots & 0 & 0 & Z_{s+1} & (1-q)r & \ldots & 0\\ & & \ddots \\ 0 & \ldots & \ldots & & \ldots & 0 & (1-q)r & Z_{n}\\ 0 & \ldots & \ldots & & \ldots & 0 & Z_{n} & 0 \end{array}\right].$$

Then *A*_*n*_(*r*) ≥ *B*_*n*_(*r*) (the inequality is componentwise) and hence *λ*_max_(*A*_*n*_(*r*)) ≥ *λ*_max_(*B*_*n*_(*r*)). Using Cauchy’s interlacing theorem, we have that the largest eigenvalue of *B*_*n*_(*r*) is larger than or equal to the largest eigenvalue of any of its principal minors. Thus,

4.5 $$\lambda_{\max}(B_{n}(r))\geq f\lambda_{\max}(G_{s})+(1-q)r,$$

and

4.6 $$\Rightarrow \lambda_{\max}(A_{n}(r))\geq 2f\cos\left(\frac{\pi }{s+2}\right)+(1-q)r.$$

By equation (2.1), we get

4.7 $$r^{*}\geq 2f\cos\left(\frac{\pi }{s+2}\right)+(1-q)r^{*}.$$

By equation (2.2), we get

4.8 $$R_{n}\leq \frac{q^{2}}{{\left(2f\cos(\pi /(s+2))\right)}^{2}}.$$

Since this holds for any \epsilon > 0 and any integer *s*, and by equation (4.4), the proof of the theorem is completed. ▪

**Proof of theorem 3.4.**

By the pigeonhole principle, there exist a subsequence of *Y*^*π*^ of length at least *n*/*d*, which consists of consecutive *X*_*i*_’s. In the proof of proposition 1, the range of parameter *h* becomes (h+1)s≤⌊n/d⌋ and we have *n*/*d* → ∞ which implies *h* → ∞ as well. The lower bound also holds due to the condition in equation (3.2). Hence, by applying the arguments used in proof of theorem 3.1, we get the result. ▪

**Proof of theorem 3.5.**

Let *ε* > 0. Consider an integer 1 ≤ *s* ≤ *n* − 1. Let $a(\epsilon )\;:=P\{Z_{1}\geq M_{Z_{1}}-\epsilon \}$. The arguments in the proof of theorem 3.1 implies that

4.9 $$r^{*}\geq \frac{2(M_{Z_{1}}-\epsilon )}{q_{L}}\cos\left(\frac{\pi }{s+2}\right),$$

with probability $P(S)\geq 1-{\left(1-{\left(a(\epsilon )\right)}^{s}\right)}^{\left\lfloor (n-1)/s\right\rfloor }\geq 1-\exp(-\lfloor (n-1)/s\rfloor {\left(a(\epsilon )\right)}^{s})$. By equation (4.9), we get

$$\begin{array}{rl} R_{n_{i}} & \leq {\left(q_{L}\right)}^{2}{\left(2(M_{Z_{1}}-\epsilon_{i})\cos\left(\frac{\pi }{s_{i}+2}\right)\right)}^{-2}\\ & ={\left(q_{L}\right)}^{2}{\left(2M_{Z_{1}}\right)}^{-2}\left(1+\frac{\epsilon_{i}}{M_{Z_{1}}}+o(\epsilon_{i})\right)\left(1+\frac{\pi^{2}}{{\left(s_{i}+2\right)}^{2}}+o(s_{i}^{-2})\right)\\ & ={\left(q_{L}\right)}^{2}{\left(2M_{Z_{1}}\right)}^{-2}(1+O(\epsilon_{i}+s_{i}^{-2})). \end{array}$$

The lower bound can be attained as in theorem 3.1 and hence this completes the proof of the theorem. ▪

**Proof of theorem 3.6.**

From equation (4.1), we have

4.10 $$\lambda_{\max}(A_{n}(r))\leq \max_{1\leq i\leq n}(X_{i-1}^{-1/2}+X_{i}^{-1/2})+(1-q_{\ell })r.$$

By equation (2.2), we have

4.11 $$r^{*}\leq \frac{\max_{1\leq i\leq n}(X_{i-1}^{-1/2}+X_{i}^{-1/2})}{q_{\ell }}$$

and

4.12 $$\Rightarrow \frac{{\left(q_{\ell }\right)}^{2}}{(\max_{1\leq i\leq n}(X_{i-1}^{-1/2}+X_{i}^{-1/2}){)}^{2}}\leq R_{n}.$$

For 1 ≤ *s* ≤ *n* − 1, let $J_{s}\in J_{s}^{n-1}$. Let *B*_*n*_(*r*) be the matrix obtained by replacing (*s* + 1) × (*s* + 1) principal minor corresponding to the indices *J*_*s*_ of *A*_*n*_(*r*) by $G_{s}\min_{i\in J_{s}}X_{i}^{-1/2}$. Thus,

$$\begin{array}{rl} \lambda_{\max}(A_{n}(r)) & \geq \lambda_{\max}(G_{s}{\text{min}}_{i\in J_{s}}X_{i}^{-1/2})+(1-q_{L})r\\ & \geq 2\cos\left(\frac{\pi }{s+2}\right){\text{min}}_{i\in J_{s}}X_{i}^{-1/2}+(1-q_{L})r. \end{array}$$

Since this holds for any choice of 1 ≤ *s* ≤ *n* − 1 and $J_{s}\in J_{s}^{n-1}$, therefore we get the upper bound given in equation (3.5). ▪

**Proof of theorem 3.7.**

Let $m_{X}\;:=\underset{c\geq 0}{\sup}\;\{P[c\leq |X|]=1\}$. We shall state a proposition that has proof similar to the proof of proposition 1.

**Proposition 4.2**

*Suppose that* ${\left\{V_{i}\right\}}_{i=1}^{n}$ *are i.i.d. random variables and are almost sure bounded. Let* *η* > 0. *Consider an integer* 1 ≤ *s* ≤ *n* *and let* *S* *be the event that there exists an index* 1 ≤ *k* ≤ *n* − *s* + 1 *such that* $V_{k},\ldots ,V_{k+s-1}\leq m_{V_{1}}+\eta$. *Then the probability of* *S* *converges to one as* *n* → ∞.

Now, using proposition 4.2 and the arguments in the proof of theorem 3.1, completes its proof. ▪

**Proof of theorem 3.10.**

Using the arguments given in theorem 3.4 and in the proof of theorem 3.7, completes the proof. ▪

**Proof of theorem 3.11.**

Let *η* > 0. Consider an integer 1 ≤ *s* ≤ *n*. Let $b(\eta )\;:=P\{Q\leq m_{Q}+\eta \}$. Using the arguments as in proof of theorem 3.7, we have

4.13 $$r^{*}\geq \frac{2\lambda_{\ell }}{\left(m_{Q}+\eta \right)}\cos\left(\frac{\pi }{s+2}\right),$$

with probability $P(S)\geq 1-{\left(1-{\left(b(\eta )\right)}^{s}\right)}^{\left\lfloor n/s\right\rfloor }\geq 1-\exp(-\lfloor n/s\rfloor {\left(b(\eta )\right)}^{s})$ . By equation (4.13), we get the upper bound. Similarly, the lower bound can be attained as in theorem 3.1 and hence, the proof of the theorem can be concluded. ▪

**Proof of theorems 3.12 and 3.13.**

The proof is based on the same approach used in theorem 3.6 and thus omitted. ▪

## 5. Discussion

Analysing the flow of particles along the tracks is of paramount importance to understand the dynamics of transport processes including the flow of biological machines like motor proteins along filaments, the evacuation dynamics, etc. Various models both deterministic and stochastic have been proposed to model the movement of particles along the lattice. The RFM is a recent area of research to rigorously analyse such processes. This is a deterministic, synchronous and continuous-time mathematical model that is an approximation of TASEP.

The RFMD is a generalized version of the RFM that models an important feature of sites having different sizes that was not incorporated in the RFM. The RFMD analyses the motion of particles in a preferred direction along a lattice through a system of nonlinear ordinary differential equations. The dynamics always converge to a steady-state density and thus implying a constant flow rate eventually. Certain types of randomness or uncertainty are always present in many nonlinear systems. An important question in this context is how the steady-state flow rate in the RFMD is affected by these fluctuations.

In this paper, we analyse the stochasticity in RFMD through the consideration of randomness in all the parameters by assuming them as random variables. Our analysis includes some closed-form theoretical results under restrictive assumptions such as rates are i.i.d. random variables. We show that, given a constant homogeneous site size, the steady-state flow rate ultimately depends on the site size and the minimal value of the random variables modelling the transition rates as the number of sites increases. This scenario also holds where the assumption on the random variables as i.i.d. is relaxed a bit. This may explain that the steady-state flow rate can be maintained in spite of some variations in the transition rates. Furthermore, we derive bounds for the steady-state flow rate in the case of a finite dimension of the RFMD having rates as i.i.d. variables and also in the case where transition rates are drawn from arbitrary but bounded random variables.

Next, we analyse the steady-state flow rate in the case of deterministic transition rates and stochastic site capacities. Similarly, we prove that given a fixed homogeneous transition rate, as the number of sites increases, the steady-state flow rate depends on the transition rate and the minimum value that the random variable modelling the site sizes attains. Our results also provide bounds on the steady-state flow rate given the general case of arbitrary site capacities. In the last and most general result, we derive bounds on the steady-state flow rate given different distributions of the transition rates or the site sizes.

In conclusion, our work provides some asymptotic results and bounds on the output of the RFMD and our observations are not dependent on the specific statistical distribution. For further research, one can develop a different approach to derive results for the convergence of the steady-state flow rate to the limiting value in the case of stochasticity in all the parameters in the RFMD. Moreover, one can analyse the steady-state flow rate in the RFMD by assuming transition rates and site capacities as dependent random variables. We believe that the results described here will be useful to analyse systems modelled through the RFMD with rates subject to uncertainties or fluctuations. For example, to analyse the performance of wireless line networks or multi-receiver diversity with random-varying connectivity.

## Data Availability

All the required data are included in the paper.
